# Linking Associations of Rare Low-Abundance Species to Their Environments by Association Networks

**DOI:** 10.3389/fmicb.2018.00297

**Published:** 2018-03-07

**Authors:** Tatiana V. Karpinets, Vancheswaran Gopalakrishnan, Jennifer Wargo, Andrew P. Futreal, Christopher W. Schadt, Jianhua Zhang

**Affiliations:** ^1^Department of Genomic Medicine, The University of Texas MD Anderson Cancer Center, Houston, TX, United States; ^2^Biosciences Division, Oak Ridge National Laboratory, Oak Ridge, TN, United States; ^3^Department of Surgical Oncology, The University of Texas MD Anderson Cancer Center, Houston, TX, United States; ^4^Department of Epidemiology, Human Genetics and Environmental Sciences, University of Texas School of Public Health, Dallas, TX, United States; ^5^Department of Microbiology, University of Tennessee, Knoxville, Knoxville, TN, United States

**Keywords:** metagenome, microbiome, unsupervised analysis, alpha and beta diversity, sparse data, Anets, qualitative data

## Abstract

Studies of microbial communities by targeted sequencing of rRNA genes lead to recovering numerous rare low-abundance taxa with unknown biological roles. We propose to study associations of such rare organisms with their environments by a computational framework based on transformation of the data into qualitative variables. Namely, we analyze the sparse table of putative species or OTUs (operational taxonomic units) and samples generated in such studies, also known as an OTU table, by collecting statistics on co-occurrences of the species and on shared species richness across samples. Based on the statistics we built two association networks, of the rare putative species and of the samples respectively, using a known computational technique, Association networks (Anets) developed for analysis of qualitative data. Clusters of samples and clusters of OTUs are then integrated and combined with metadata of the study to produce a map of associated putative species in their environments. We tested and validated the framework on two types of microbiomes, of human body sites and that of the *Populus* tree root systems. We show that in both studies the associations of OTUs can separate samples according to environmental or physiological characteristics of the studied systems.

## Introduction

The rare low-abundance microbial species, which have been referred to as the “rare biosphere” (Sogin et al., 2006), have attracted increasing attention in the recent literature because of their unknown ecology and potential evolutionary and ecological importance (Youssef et al., 2010; Pedros-Alio, 2012; Coveley et al., 2015; Lynch and Neufeld, 2015; Sharon et al., 2015; Jousset et al., 2017). Although sequencing errors and undersampling of OTUs may contribute to extent of the “rare biosphere,” the advent of new bioinformatics tools (Schloss and Westcott, 2011; Preheim et al., 2013; Edgar and Flyvbjerg, 2015; Sharon et al., 2015; Callahan et al., 2016) as well as experimental and technological approaches (Jousset et al., 2017) are increasingly compelling of the presence and complexity of these rare taxa. Biological explanations (Pedros-Alio, 2012; Coveley et al., 2015; Lynch and Neufeld, 2015; Jousset et al., 2017) and other factors, such as poor taxonomic resolution of short reads, especially for closely related species or those poorly represented in the genomic database, incomplete or inadequate sampling, dispersal limitation, spatial and temporal partitioning of the environment, and the nestedness of ecological mutualistic networks, may contribute to such results (Bascompte et al., 2003; Youssef et al., 2010; Rosindell et al., 2011; Unterseher et al., 2011; James et al., 2012; Mi et al., 2012; Pedros-Alio, 2012; Suweis et al., 2013).

The numerous rare OTUs are a typical output of 16S rRNA amplicon sequencing studies, especially those with many and diverse samples. The resultant sparse datasets present a challenge for common statistical tools. The data matrix produced by such studies are usually comprised of species-like groups (rows) and their abundances calculated as the number of sequencing reads representing each species across multiple samples (columns). The species-like groups are typically inferred by a conventional aggregation of sequences into OTUs based on a sequence identity threshold or, in more recent work, by amplicon sequence variants (ASVs) (Callahan et al., 2016; Callahan, 2017). In both cases, most species-like groups could be representative of species-specialists; they are not only low in abundance in a given sample, but are also rare across samples and environments. Known computational tools for analyzing the sparse data often address the sparsity problem by filtering out very rare species or by collapsing species to a higher-level hierarchy. Although the aggregation reduces sparsity (dominance of zeros in the dataset) of the data, the OTUs-level insights into the structure of microbiome will be lost. By excluding the rare OTUs, such as those found in less than 30% of samples, we also may lose information. It is not clear how extensive this loss might be.

In addition to sparsity, the 16S rRNA gene sequencing data have other challenges including their compositionality and dimensionality (essentially greater number of OTUs than the number of samples). The data compositionality means that we don’t know the real OTU abundances and have to deal with proportions of species relative to their sum in each sample. Several methods have been proposed to address the challenges (McMurdie and Holmes, 2014; Tsilimigras and Fodor, 2016). The most recent methods proposed to infer species–species relationships from the 16S rRNA amplicon datasets include Compositionality Corrected by REnormalization and PErmutation (CCREPE) (Faust et al., 2012), metagenomeSeq (Paulson et al., 2013), Sparse Correlations for Compositional data (SparCC) (Friedman and Alm, 2012), a mixture model framework (McMurdie and Holmes, 2014), SParse InversE Covariance Estimation for Ecological Association Inference (SpiecEasi) (Kurtz et al., 2015), and gCoda (Fang et al., 2017). Each of the tools addresses dimensionality and compositionality challenges of the datasets using different computational approaches. The cumulative sum scaling normalization and the zero-inflated Gaussian distribution mixture model are used in metagenomeSeq to account for biases resulting from under-sampling when selecting the differential abundant OTUs. The log-ratio transformation and the variance are used in SparCC to overcome compositionality of the data. The data dimensionality and compositionality are even more efficiently addressed by SpiecEasi and gCoda using the data transformation borrowed from the compositional data analysis and then inferring the interaction graph from the transformed data by neighborhood selection or by sparse inverse covariance selection.

All abovementioned tools, however, analyze the OTU table after filtering out most rare OTUs (Supplementary Figures S1A–D). In case of SparCC, the filtering is the most stringent because the algorithm employs log-transformations of the read counts. The basic assumption of the approach is that all OTUs are present in the dataset; therefore small values must be assigned to undetected OTUs to include them in the analysis. The percentage of rare OTUs may be even greater in studies with large number of samples or when sampling takes place in more diverse environments, such as the Human Microbiome Project (HMP) dataset and the *Populus* Root Microbiome (PRM) dataset (Supplementary Figures S1E,F). In the study we have made an attempt to explore the biological role of the rare low-abundance OTUs in these two environments using existing data from Human body sites (2012) and from *Populus* roots (Shakya et al., 2013). To reduce the burden of filtering for the rare OTUs and overcome the problem of compositionality we treat the OTUs as qualitative variables and apply an analytical tool specific for analysis of such datasets.

## Results

### Approach

**FIGURE 1:**
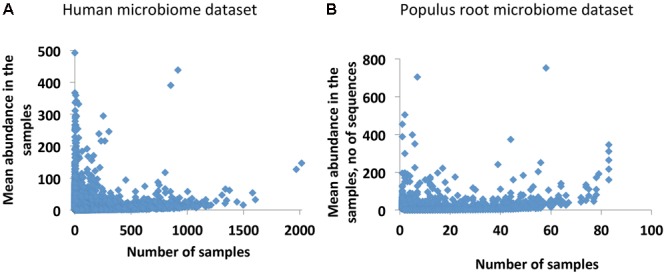
Occupancy–abundance relationship. **(A)** Human Microbiome Project (HMP) dataset (43140 OTUs × 2910 Samples). **(B)** *Populus* Root Microbiome (PRM) dataset (24434 OTUs × 83 Samples).

Our initial analysis of the Human and *Populus* microbiome datasets reveals that both datasets are in agreement with the well-known occupancy–abundance relationship (Gaston, 1996), which positively links the species abundances and the number of sites/samples they occupy. We find that in both datasets, OTUs that are more common across samples are also more abundant, and rare OTUs across samples are usually less abundant (**Figures 1A,B**). Notably, the number of common abundant OTUs is extremely small in the datasets. Considering this observation we decided to treat the rare OTUs as qualitative data by replacing the putative species abundances with the presence/absence call (0/1 values). Although in this approach we lose information on abundances, at the same time, the resulting dataset will not be compositional. In addition, we get the chance to transform the data to collect additional statistics on co-occurrences of species with each other and to quantify interdependencies of the species. The quantification is based on an assumption that rare OTUs (putative species) are associated because they are dependent upon one another in each studied environment. They may be dependent metabolically, when metabolites produced by one species are consumed by another species. They also may have similar optimal growth conditions or offer complementary functions to support microbial community as a whole (Jousset et al., 2017). All these factors may lead to co-occurrences of the rare OTUs in the samples. We quantify the co-dependence of OTUs by calculating a co-occurrence profile of each OTU with all other OTUs in the data and by interrogating similarities of the emerged profiles for each pair of OTUs. We performed the calculations by applying a previously developed statistical tool, Association Network (Anets) (Karpinets et al., 2012), used for discovering of associations in qualitative datasets^1^ and refer to the resultant network as Anets-OTUs.

**FIGURE 2:**
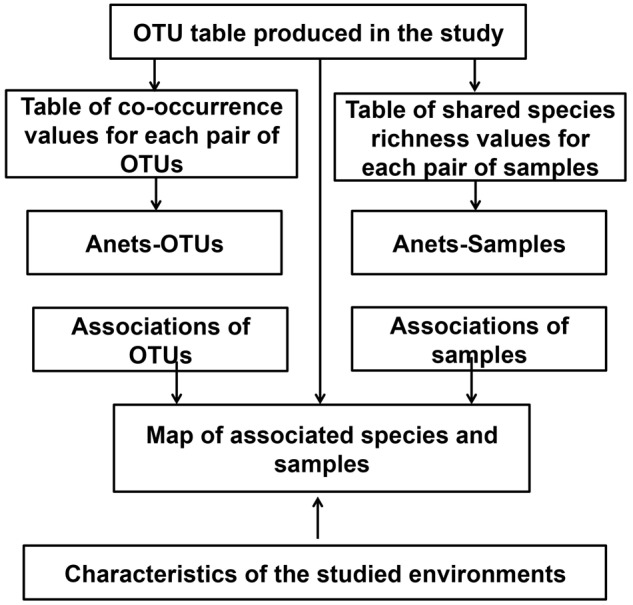
Computational framework used in the study to explore associations of rare species.

In addition to that Network, we also build the network of samples, Anets-Samples, using the same algorithm. By combining both networks we produce a map where associated OTUs and associated samples are clustered according to their presence/absence. This map can be further compared with characteristics of the studied environments. An overview of this computational framework is shown in **Figure 2** and details of the implementation are provided in Supplementary Data Sheet S1.

**FIGURE 3:**
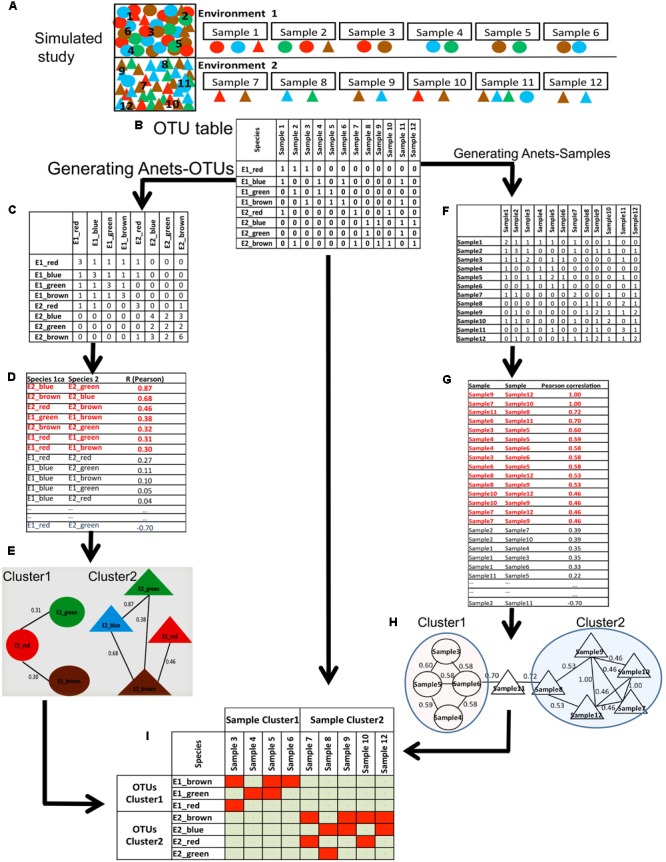
Generating Anets-OTUs using the simulated study. **(A)** A simulated study of two synthetic microbial communities: four species shown by colored (red, green, blue, brown) circles (Community 1), and four different species shown by colored (red, green, blue, brown) triangles (Community 2). The same color of the species indicates their close taxonomic relationship. To introduce noise in sampling, two species from the second community were added to the first community, and one species from the first community was added to the second community. Six samples were taken to identify species in each community and to generate an OTU table with the species abundances. **(B)** OTU table of the simulated study. **(C)** The table of co-occurrences for each pair of OTUs. Values of the table show the number of samples where each pair of species co-occurs. **(D)** Pair-wise similarities of the co-occurrence profiles for each pair of species. Red colored associations were used to generate Anets-OTUs. **(E)** Anets-OTUs. **(F)** The table of the shared species richness for each pair of samples. Values of the table show how many OTUs are shared for each pair of samples. **(G)** Pair-wise similarities of the shared species richness profiles for each pair of samples. Red colored associations were used to generate Anets-Samples. **(H)** Anets-Samples. **(I)** A map of the associated species and samples.

We also used a simulated dataset (**Figure 3A**) to illustrate and explain computations underlying the proposed framework. In this study, we have two synthetic microbial communities with four associated species (circles) in the first community and four associated species (triangles) in the second community. Species in each community are co-dependent, and therefore more often co-occur in their parent environment. We made 12 random samples of species from the communities and organized the sampling results as an OTU table (**Figure 3B**) with species/OTUs in rows and samples in columns. All species identified in the samples are rare; they are found only in 2–5 out of 12 samples. Thus, we replaced the species abundances with the presence/absence (1/0) values.

#### Association Network of Species

To generate the Anets-OTUs we first transform the OTU table to produce a new table where rows and columns consist of OTUs and each cell shows the number of samples where two OTUs co-occur in the data (**Figure 3C**). The transformed table, therefore, gives us the co-occurrence profiles for each OTU with the rest. We further use these profiles to infer pair-wise associations of the OTUs (**Figure 3D**). Although the input of the approach is OTU table with 1/0 values instead of counts, the statistics collected in the transformed table produces continuous variables. The Anets program provides three options to quantify the pair-wise similarities of the profiles. The options include Spearman correlation (default), Pearson correlation, and cosine (Jaccard index). While alternative similarity metrics may be appropriate for particular datasets, in these studies we found that the Pearson correlation coefficient was most robust for identifying association networks. We calculate the Pearson correlation to measure similarity of the profiles for each pair of OTUs and consider the OTUs associated if the correlation coefficient R > = 0.30. The selected pairs of OTUs predict the network (Anets-OTUs) of seven species with seven associations separated into two clusters/communities (**Figure 3E**). The species inferred by the Anets-OTUs in each cluster correspond to two communities provided in the mock study (**Figure 3A**). The algorithm did not recover only one species from the Environment 1 of the study.

While, the calculations described in this small illustrative dataset can be implemented in Excel, in case of real datasets, with many samples and OTUs, the calculations can be performed using the Anets program (Karpinets et al., 2012). The program also calculates the *p*-value for each association using the Monte-Carlo simulation. The associated species, therefore, can be selected using a *p*-values threshold. The Anets-OTUs produced for the mock study is small and doesn’t require clustering. For the real dataset, different algorithms and software tools can be used to cluster the network as described in Supplementary Data Sheet S1.

#### Association Network of Samples

A similar algorithm was used to generate the associations of samples (**Figures 3F–H**). In this case we transform the OTU table to produce a new table where both rows and columns consist of samples and each cell represents the number of shared OTUs for each pair of samples. The ecological interpretation of the number is the shared species richness for a pair of samples. We consider two samples associated if they have a similar profile of the shared species richness values across all samples in the dataset. Such indirect similarity can establish an association between each pair of samples even if the majority of species in the samples are not common. Computationally, the algorithm generating the Anets-Samples (**Figures 3F–H**) is similar to the algorithm of the Anets-OTUs (**Figures 3C–E**). As before, the transposed table is used to compute profiles of shared species richness values for the samples (**Figure 3F**) followed by estimation of pair-wise correlations (**Figure 3G**) and clustering (**Figure 3H**). As we can see in the **Figure 3H**, the clustering recovers associations among 9 out of 12 samples in the illustrative study. The final step of the framework is an integration of the results obtained by Anets-OTUs and Anets-Samples by building a presence/absence map of the associated species and samples (**Figure 3I**).

### Applying the Approach to Experimental Datasets

In order to test our methodology, we employed the described framework to analyze two well- established and published experimental datasets from a study of Human Microbiome Project Consortium, 2012 and from a study of the PRM (Shakya et al., 2013). In each of these datasets, 16S or 28S rRNA amplicon sequencing was used to profile the microbiome in different environments. By applying our methodology in an unsupervised manner to build a map of associated OTUs and samples, we were able to test how well the inter-sample associations reproduced their observed phenotype in the environment, with the added advantage of studying associations of rare OTUs underlying the grouping of samples.

#### *Populus* Root Microbiome

The dataset (Shakya et al., 2013) includes 2999 fungal OTUs and 24435 bacterial OTUs identified in 84 samples taken in May and in September from two geographical locations, Tennessee (TN) and North Carolina (NC) associated with the roots of Eastern Cottonwood (*Populus deltoides)* trees at along two different rivers. The study also collected a set of soil properties and host characteristics for each of the 23 sampling locations; we used these metadata to examine their relationships with the associations of samples discovered by the Anets-Samples.

**FIGURE 4:**
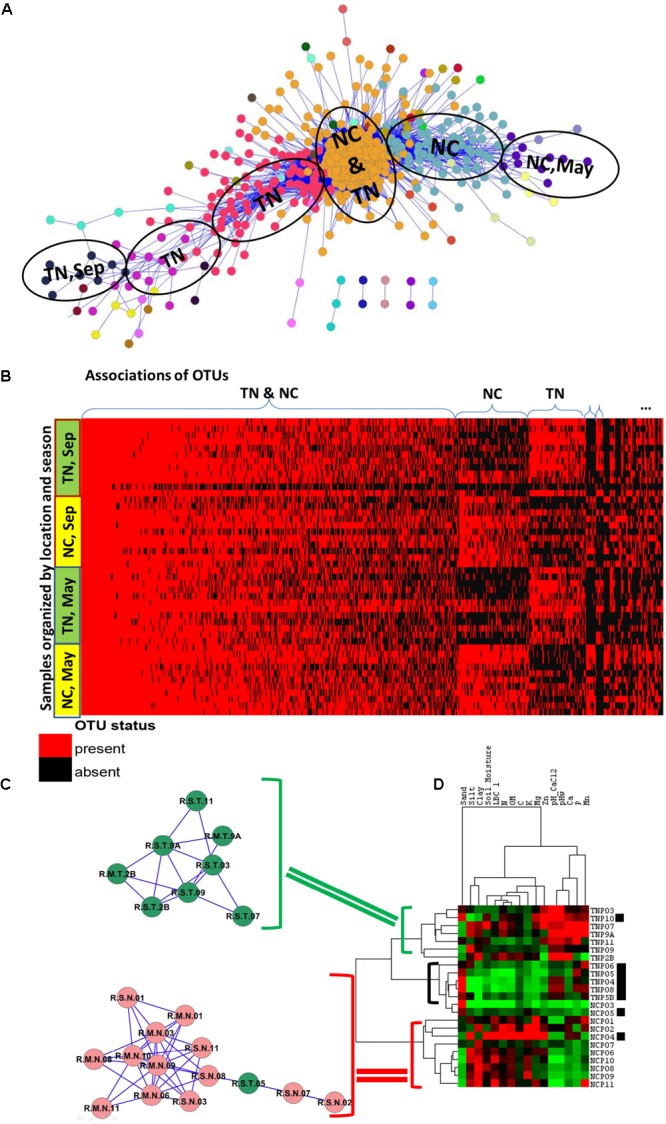
Associations of rare species and samples in PRM study. **(A)** Communities of associated fungal and bacterial OTUs discovered by the Anets-OTUs algorithm in rhizoshpere of *Populus deltoides*. Nodes in the network indicate OTUs and edges indicate pair-wise association between them. The node color shows the community (cluster) assignment inferred by clustering. **(B)** Presence–absence map of the associated OTUs; the cell color is red if OTU is present in the sample and it is black if OTU is absent. OTUs are grouped according to the microbial communities inferred by Anets-OTUs and sorted by mean abundance; samples are grouped according to clusters inferred by Anets-Samples and sorted by the shared richness. **(C)** Two associations of *Populus* rhizoshpere samples with the shared species richness revealed by Anets-Samples; color indicates samples taken in NC (red) and in TN (green). **(D)** Hierarchical clustering of the soil properties; brackets indicate three cluster of soil samples with distinct soil properties: green bracket indicates the cluster of soil samples that correspond to the association of rhizosphere samples in TN, red bracket indicates the cluster of soil samples that correspond to the association of rhizosphere samples in NC, black bracket and black squares indicate samples that don’t found as associated by Anets-Samples.

Examination of the OTU table from the study reveals that common species (found in ∼60% of samples) or generalists in *Populus* root are represented by only 61 OTUs, or 0.22% of total number of OTUs in the dataset. As expected, the majority of OTUs had low-abundance and was rare (**Figure 1B**). After applying the Anets-OTUs algorithm to the OTU table we found six large associations of OTUs (*p*-value < 0.05). A further enrichment analysis (see section “Materials and Methods”) attributed each association to a location, TN and NC, and to a sampling season, May or September (**Figure 4A**). This analysis revealed that communities of low-abundance OTUs, were underlying groups of samples based on known environmental factors from the study. To further confirm the grouping we built a heat map of the associated OTUs (horizontal axis) across all samples (vertical axis) organized by the geographical location and season and sampling (**Figure 4B**). While, it can be appreciated that many rare Anets-OTUs are present across all samples, some of them often co-occur in samples from a particular location or a season. The largest microbial association includes OTUs found in *Populus* rhizosphere in any season and in any location. Some associations are more common for TN or NC, and some associations are more common in September or May. This pattern suggests a tight link between the identified associations of the rare OTUs and a particular environmental factor. We noticed, for example, that a fungal OTU representing the genus *Inocybe* was found only in the NC cluster. Indeed, species of the genera have been tied to their environments rather than their hosts more than other fungal species (Cripps, 1997). Our results are consistent with this experimental observation; they also indicate that the other fungal genera in the cluster, such as *Ceratobasidium*, have similar biological characteristics.

The analysis confirms that clustering at low taxonomic levels may be crucial in discriminating different environments. We find that although OTUs in each of the associations often belong to the same phyla, they are more distinct at lower taxonomic levels, such as order (Supplementary Tables S1A,B). For example, microbial communities of *Populus* roots in both locations, TN and NC, include phylums *Proteobacteria* with less number of OTUs in NC (Supplementary Table S1A). At the level of order, however, the *Proteobacteria* in NC had greater richness (10 orders) when compared with TN (seven orders), and included *Rhodocyclales, Syntrophobacterales, Rhodobacterales*, and *Burkholderiales* orders that were not observed in TN. Microbial communities in both locations, TN and NC, also included numerous species from phylum *Acidobacteria.* The microbial community in TN, however, was dominated by the order Solibacterales; this taxa, however, was not found in NC. This example clearly demonstrates that by analyzing the dataset at the level of OTUs and collapsing them after linking their associations to environments may be a better strategy for exploration of subtle difference among microbiomes in similar environments.

By applying the Anets-Samples algorithm to the OTU table we revealed two distinct clusters of samples in the PRM dataset (**Figure 4C**). Within each cluster, all samples had similar profiles of the shared species richness across all samples (*p* < 0.01). Furthermore, there was a clear association with metadata of the study, with the first cluster representing a subset of samples from TN, and the second cluster representing a subset of samples from NC. Eight samples did not associate with either cluster. These results mirror the results of Shakya et al. (2013) that used variance partitioning of transformed datasets to show that watershed (TN vs. NC), season, and sampling site within a watershed, respectively, had the greatest effect on community structure followed by other factors. To determine other environmental factors contributing to the separation of samples in two clusters we examined the variance partitioning of the bacterial OTUs within each cluster with respect to host and soil properties, geographic locations, seasons, and diversity of corresponding fungal community. The analysis was performed the same way as in the original study (see section “Materials and Methods”). A large proportion of variance (67.8%) of the bacterial OTUs across all samples was unexplained in the original study, whereas only 9% of variance was explained by soil properties. In contrast, among the samples that were selected by the Anets-Samples as significantly associated, only 25% of variance remained unexplained, while the greatest proportion of the variance (30.1%) was attributed to the studied soil properties (Supplementary Figure S2). The expected proportion of the variance estimated by the permutation test, via a random selection of the same number of samples, would be only 19%.

To examine the effect of soil on the separation of samples in more detail we hierarchically clustered 16 soil properties measured in the study and found that two associations discovered by the Anets-Samples in *Populus deltoides* rhizosphere (**Figure 4C**), correspond to two distinct soil clusters inferred from the soil properties (**Figure 4D**). This relationship was not found in the original study and again suggesting the importance of rare microbial species for differentiating subtle environmental conditions in addition to the traditional methods that more heavily weight species abundance and dominant taxa. In case of PRM we observe that a set of TN samples found as associated by Anets share relatively greater Zn, Mn, and Ca contents in the soil and a greater soil pH. A set of associated NC samples share relatively low values of these soil characteristics. Those samples, either from TN or NC, that are not identified by Anets-Samples as significantly associated, have a variable content of the soil properties as well as relatively greater sand content and lower clay and organic matter contents than the associated samples. The results point to the soil properties as a crucial factor underlying similarity of microbial communities in *Populus deltoides* rhizosphere.

#### Microbiomes of Human Body Sites

**FIGURE 5:**
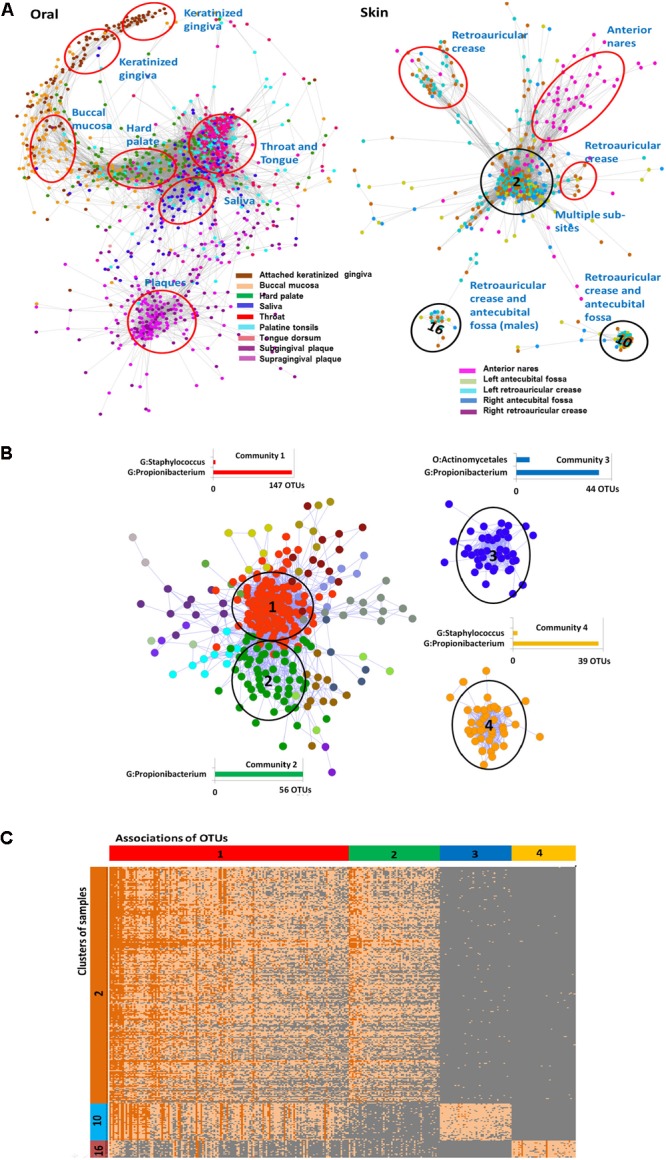
Associations of rare species and samples in the HMP study. **(A)** Associations of oral and skin samples. Samples in the networks are represented by filled circles colored according to the sampling sub sites in the HMP study. Edges between circles indicate significant association between samples in terms of the shared species richness. Red and black ovals label associations predicted by clustering of the Anets-Samples. Name of each cluster was inferred by the enrichment analysis as described in Section “Materials and Methods.” Black ovals indicate clusters (2, 10, and 16) that were further analyzed by the Anets-OTUs algorithm. **(B)** Associations of rare species discovered by Anets-OTUs in samples comprised clusters 2, 10, and 16. Small components of the network are not included. OTUs are represented by nodes (filled circles) where color indicates different clusters inferred by Markov clustering. The largest clusters are referred as communities. Edges between nodes represent significant associations (*p* < 0.001) between a pair of OTUs. They are labeled by black ovals and have associated bar charts showing the number of OTUs from most abundant taxonomic ranks labeled as G (*Genus*) and O (*Order*). **(C)** Heat map of abundances (in terms of sequencing reads) of associating microbial OTUs (horizontal axis) in three distinct clusters of samples (vertical axis) collected from the human skin. OTUs are grouped according to the microbial communities inferred by Anets-OTUs and sorted by mean abundance; samples are grouped according to clusters inferred by Anets-Samples and sorted by the shared richness. Each cell shows the number of OTU reads. Color of cells in the map shows the number of reads representing the OTUs in the sample: 10 reads or more (dark orange), from 1 to 10 reads (light orange), and not represented by reads (gray). Cluster IDs indicated in **(A,B)** are shown in vertical and horizontal bars of the heat map respectively.

The HMP dataset has been characterized in several publications (Faust et al., 2012; Project, 2012; Aagaard et al., 2013) and includes samples obtained from 18 different body sites of 180 healthy men and women. As noted before (**Figure 1A**), the majority of OTUs in the dataset is rare and has low-abundance. Considering the large size of the OTU table produced in the study we started the analysis with the construction of the Anets-Samples (**Figures 3F–H**) to find associations (clusters) of samples with similar profiles of the shared species richness and to discard samples-outliers. Most samples (74%) in the dataset were found to be associated (*p*-value < 0.01) with at least one other sample in the network. Visualization and clustering of the network using the Markov clustering algorithm (MCL) (Van Dongen, 2008) revealed seven large disconnected component and 206 clusters (Supplementary Figure S3). We next used an enrichment analysis (see section “Materials and Methods”) to annotate the inferred clusters by sample metadata (sex of the human subject, body site, and sub-site) and to assign significantly enriched body sites and sub-sites to the clusters. **Figure 5A** shows components of the network comprised of oral and skin samples colored according to sub-sites. Samples that belonged to a particular subsite tended to cluster together according to the Figure and to the enrichment analysis. Thus, the Anets-Samples allowed us to predict origin of samples from different oral sub-sites, such as keratinized gingiva, buccal mucosa, hard palate, saliva, throat, and tongue. There were also several distinct associations of samples originated from multiple skin subsides. Interestingly, one association of samples (cluster 16 in **Figure 5A**) was comprised of male human subjects.

We further focused the analysis on 314 skin samples that represent three distinct, disconnected in the Anets-Samples, clusters labeled by black ovals in **Figure 5A**. To reveal communities of microbial OTUs discriminating these clusters we built the Anets-OTUs using, as input, an OTU table comprised of these 314 samples in columns and 43140 OTUs in rows. The generated Anets-OTUs included 412 associated OTUs (*p*-value < 0.001); and subsequent clustering of the network revealed four major microbial communities (**Figure 5B**). The enrichment analysis showed statistically significant links between the communities and the Anets-Samples clusters (Supplementary Table S2). The map generated from the initial dataset by extracting abundance values of the associating OTUs further confirmed the links (**Figure 5C**). Importantly, the three distinct clusters of samples, originated from skin of different human subjects, have significant differences in microbial communities at the OTU level, although most OTUs contributing to the difference belonged to the genus *Propionibacterium.* Indeed, microbial community 1 comprised of OTUs of the genus *Propionibacterium* (**Figure 5B**) was significantly enriched in Anets-Samples clusters 2 and 10 (**Figure 5A**), but not in Anets-Samples cluster 16 (**Figure 5A**). Microbial community 2 comprised of a distinct set of OTUs from the same genus (**Figure 5B**) was significantly enriched only in Anets-Samples cluster 2 (**Figure 5A**). The third microbial community comprised of OTUs of the genera *Propionibacterium* and *Actinomycetales* (**Figure 5B**) was enriched in Anets-Samples cluster 10 (**Figure 5A**), and the fourth microbial community (OTUs from the genera *Staphylococcus* and *Propionibacterium*) was enriched in Anets-Samples cluster 16 comprised of male human subjects. The *p*-value 0.01 (Fisher exact test) was used as the significance threshold in the enrichment analysis. Thus, the OTU level clustering was important to discriminate microbial communities of the clustered samples.

### Validation of the Anets Algorithm

We use 1250 oral samples of HMP to investigate the robustness and limitations of the Anets algorithm, to compare it with other methods and to explore potential biases and confounding factors.

#### Library Size as Potential Confounding Factor

The Library Size (LS) affects the number of identified rare species and, therefore, may introduce a technical bias in the OTU table if there are significant differences in LSs among studied environments. We explore this affect using known annotations of oral samples by subsites. Specifically, pair-wise comparisons were performed among all the subsites in terms of the library size and then in terms of the number of rare OTUs. We find that log-transformed values of the library size in the oral samples have a normal distribution (Supplementary Figure S4). Significant differences between average values (Wilcoxon test) were observed for 2 out of 15 pair-wise comparisons (Supplementary Figure S5), and only for one comparison, “Tongue dorsum” versus “Hard palate,” the difference in LS is also associated with the significantly different number of rare species (Supplementary Table S3). In general, most rare OTUs are the least abundant and the mean number of such OTUs is significantly different in 60% subsite pairs (Supplementary Figure S6 and Supplementary Table S3). When we consider less rare OTUs we find a significant increase in the mean abundance of the OTUs (Supplementary Figure S6) and significant decrease in the % of subsite pairs that are significantly different in terms of the number of rare OTUs, from 60 (occupancy threshold 1%) to 40, 20, and 13% (occupancy threshold 5, 10, and 25%, respectively) (Supplementary Table S3). According to the results, the LS may be a confounding factor in the analysis of rare OTUs, although the different LS doesn’t necessary translate to different number of rare species, at least for oral subsites. There is a clear trend for oral subsites to be less different in terms of the number of rare OTUs when we increase the occupancy threshold. This trend, however, doesn’t associate with different LSs of the subsites.

#### Importance of Rare OTUs for Anets-Samples Construction

**FIGURE 6:**
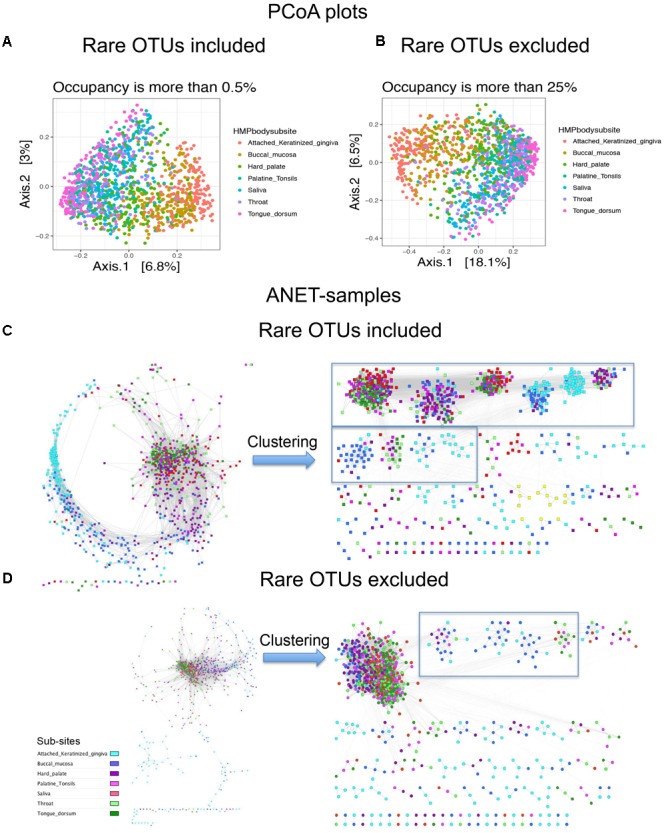
Principal coordinates analysis (PCoA) plots and Anets-Samples for oral samples with or without rare OTUs. **(A)** PCoA plot generated by including rare OTUs. **(B)** PCoA plot generated by excluding rare OTUs. **(C)** Anets-Samples generated by including rare OTUs. **(D)** Anets-Samples generated by excluding rare OTUs. Large clusters (more than 10 samples) are bordered by rectangles.

We further explore how important rare and common taxa for correct grouping of samples. We separated species identified in 1250 oral samples to two groups, rare (occupancy is between 0.5 and 25% samples) and common (occupancy > 25%). Then we generated three OTU tables; comprised of only rare OTUs, rare and common OTUs, and only common OTUs. We find that considering only rare OTUs we reduce the resolution of the Principal coordinates analysis (PCoA) plot (Supplementary Figure S7A). In case of Anets-Samples (Supplementary Figure S7B), we actually increase the resolution and were able to detect a batch effect among oral samples. The effect was probably masked by the presence of common species because we didn’t observe the effect if we use OTU table with only common OTUs (**Figure 6D**) or with common and rare OTUs (**Figure 6C**). In spite of the batch effect, the grouping of samples within the large batch (Supplementary Figure S7B) was consistent with the studied oral subsites, although not as evident as for Anets-Samples based on a combined set of rare and common OTUs (**Figure 6D**). The PCoA plots generated for OTU tables by including or excluding the rare OTUs were rather similar (**Figures 6A,B**) suggesting that we will not significantly effect the interpretation of the results by excluding rare species in the PCoA. However, by excluding the rare species when building Anets (**Figure 6D**), we essentially decrease our chance to cluster samples according to subsides (**Figures 6C,D**, right sides) and also decrease the number of associated samples (*p* > 0.05) from 1082 (87%) to 981 (78%). The results demonstrate high sensitivity of the Anets algorithm to signals from both, rare and more abundant, OTUs. The result is not surprising. To build Anets we have to collect additional statistics on co-occurrence of species with the rest and on the shared species richness to establish the pair-wise associations in Anets-Samples and in Anets-OTUs. By excluding some species, either less abundant or more abundant, we loose information important for the analysis and impair the results. Building Anets after filtering common species, however, may allow us to see biases obscured by the presence of common taxa.

#### Topological Differences Between Networks Generated Using Anets and Unweighted UniFrac Distances

**FIGURE 7:**
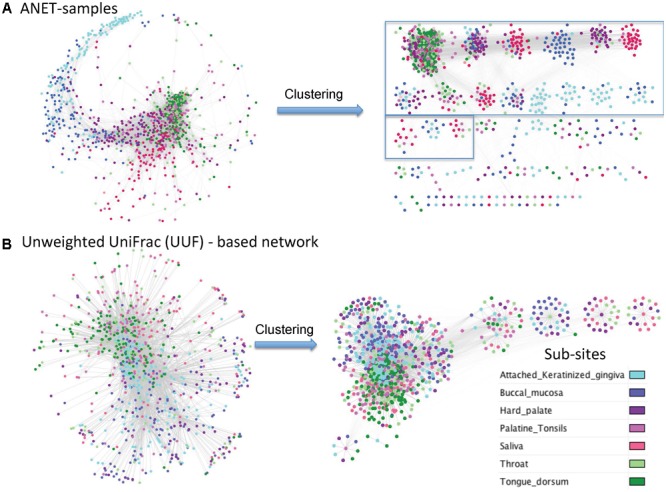
Networks of oral samples and their clustering by the Markov clustering algorithm (MCL) with the same parameters. **(A)** The network was generated using Anets-Samples algorithm. The large clusters (more than 10 samples) are bordered by rectangles. **(B)** The network was generated using Unweighted UniFrac (UUF) distances as measure of pair-wise similarity of the samples (nodes) with the threshold 0.98.

UniFrac is widely used distance metric incorporating phylogenetic information to compare microbial communities. All taxa, common and rare, are included in calculation of the distance. The metric, therefore, may be an alternative way to construct the network of samples by incorporating the phylogenetic signals from rare species. We have compared the network of samples generated by Anets with those based on the Unweighted UniFrac (UUF) distances. The ‘phyloseq’ package (McMurdie and Holmes, 2013) was used to calculate the UUF distance for each pair of oral samples. Two networks were generated with thresholds for the distance to be equal 0.95 and 0.98. We chose these thresholds because we find it difficult to break the UniFrac-based networks into clusters because of low clustering coefficients and high centralization if compared with the Anets-Samples (Supplementary Table S4). We could increase the clustering coefficient and reduce centralization by increasing the distance measure but it also reduced the number of nodes in the UUF network. Using a looser threshold (0.95) we had 1243 nodes that were vastly interconnected by 68284 edges into one large cluster (Supplementary Figure S8). By increasing the distance threshold to 0.98 we generated a network with 868 samples and 6457 edges and a greater clustering coefficient (**Figure 7B**). The generated clusters, however, were not as consistent with the annotation of subsites as in case of Anets-Samples network (**Figure 7A**). Although in general all three networks showed the same trend of separation of subsites ‘keratinized gingiva’ and ‘bunccal mucosa’ from ‘saliva,’ ‘tongue dorsum,’ and ‘throat,’ it was easier to cluster the Anets-based network, and, importantly, many large clusters in the Anets network were enriched with samples originated from the same subsite (**Figure 7A**, right side). The comparison reveals a distinct topology of the Anets network if compared with UUF-based networks and a better association of the topological structure with oral subsites. The more centralized topology of the UUF-based network may be suitable for a global overview of the samples. The Anets-based network may perform better if we want a greater level of detail and more granularity in grouping the samples.

#### Robustness of the Anets Algorithm

**FIGURE 8:**
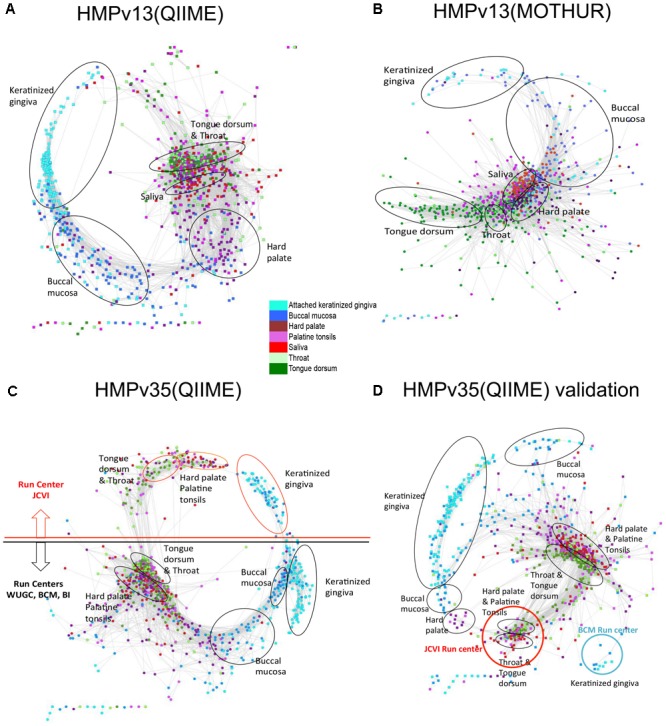
Anets-Samples generated for different OTU tables comprised of oral samples. **(A)** OTU table generated by QIIME pipeline from sequencing of 16S rRNA gene variable regions 1–3. **(B)** OTU table generated by MOTHUR pipeline from sequencing of 16S rRNA gene variable regions 1–3. **(C)** OTU table generated by QIIME pipeline from sequencing of 16S rRNA gene variable regions 3–5. **(D)** OTU table generated by QIIME pipeline from sequencing of 16S rRNA gene variable regions 3–5 of a distinct set of oral samples.

Several different factors including sampling strategy and sample handling, the choice of universal 16S rRNA gene PCR primers, DNA extraction methods, amplification artifacts, such as chimeras, and computational methods employed to produce the OTU table from sequencing reads may contribute to different results in the 16S rRNA gene profiling studies. All of them can affect the number of rare species and the produced Anets. To evaluate the robustness of the algorithm we explore changes in the structure of Anets based on OTU tables constructed by different processing pipeline, by different 16S rRNA gene variable region for sequencing, and by a different subset of oral samples. Namely, we consider three different OTU tables produced for oral samples by two commonly used 16S rRNA amplicon data processing pipelines, MOTHUR (Schloss et al., 2009) and QIIME (Caporaso et al., 2010) that utilize different algorithms to construct the OTU table. The former OTU table was produced by a high quality-filtering MOTHUR pipeline (Schloss et al., 2011) with low overall chimera rate. The formation of the chimeric sequences is a well-known factor contributing to erroneous OTUs and to overrated species richness (Ashelford et al., 2005). We also compared OTU tables generated by QIIME pipeline from sequencing of 16S rRNA gene variable regions 1–3, referred as HMP v13 (Q), and variable regions 3–5, referred as HMPv35(Q). These three OTU tables were generated for the same subset of 1250 oral samples. In addition, we included an OTU table (QIIME pipeline, v35) produced for a different subset of 1025 oral samples in the comparison. We refer to the table as HMPv35(Q) validation. The tables were downloaded from the NIH Human Microbiome Project websites and were comprised of different number of OTUs, from 8640 OTUs in HMPv13(M) to 26399 OTUs in HMPv35(Q) Validation. Most OTUs (95–97%) in the tables were rare OTUs (found in less than 25% samples). The Anets-Samples was generated for each OTU table and visualized by Cytoscape using the same parameters. Comparison of the produced networks reveals not only their similar statistical characteristics (Supplementary Table S5), but also a similar trend in grouping of samples among subtypes (**Figure 8**). The MOTHUR and QIIME networks, however, were surprisingly different in their ability to separate different subsites (**Figures 8A,B**). The MOTHUR network performed well in separating tongue dorsum and throat from other subsites, but not as good in separating keratinized gingiva and buccal mucosa, while the QIIME v13 network performed better in separating keratinized gingiva and buccal mucosa from other subsites, and not as good for tongue dorsum and throat. The difference persists when we run Anets with different parameters. An interesting symmetrical structure, related to the batch effect, was revealed in the Anets-samples produced for OTU table HMPv35(Q) (**Figure 8C**). The upper part of the network represents samples sequenced by J. Craig Venter Institute (JCVI) and the lower part representing samples sequenced by other sequencing centers. Importantly, each side of the network demonstrated similar grouping of samples into subtypes regardless of the batch affect. The network generated for a different subset of oral samples, HMPv35(Q) Validation, reveal a similar batch effect with separation of samples into subsites within each batch. Based on the results we conclude that the Anets algorithm recover similar groupings of samples from OTU tables produced by two commonly used 16S rRNA amplicon data processing pipelines regardless of the observed batch effects and type of sequencing (v13 or v35) as well as from an OTU table comprised of different samples from the same environments.

## Discussion

In this proof of concept study we aimed to demonstrate the use of the Anets-based computational framework for linking associations of rare OTUs to their environment. Results of the study demonstrate that a combination of the Anets-OTUs and Anets-Samples has a potential to serve as a powerful unsupervised methods for discovering relationships and associations of rare species from phylogenetic marker gene datasets used in microbiome studies. Applying the framework to analyses of microbiomes in *Populus* roots and on Human body sites we were able to reproduce associations of samples in these complex environment and associations of species that were consistent with the existing metadata and the analyses described in the previous literature. In case of Human microbiomes we were able to identify associations of co-dependent rare OTUs and link them to sub-sides of the human body. Similar observations were reported by Ding and Schloss (Ding and Schloss, 2014) using the Dirichlet multinomial mixture models (Holmes et al., 2012).

An important observation from the analysis of *Populus* and Human microbiomes by the approach is a close link between the rare microbial OTUs and specific environmental conditions. To explain the importance of rare putative species for classification of the environments we propose that the high-abundance OTUs are common among sampled environments because the environments have some common conditions stimulating outgrowth of the same putative species. The rare low-abundance OTUs are rare because each of these environments also has some specific conditions or microenvironments. These specific microenvironmental conditions may stimulate the growth species represented by rare OTUs. Although they are rare, they may be crucial for recovering the micro-environmental differences in microbiomes of the environments. It is possible that these rare OTUs, therefore, may be a better computational target for quantification of subtle differences among most variable properties of the environments, and their presence/absence pattern can be used for additional comprehensive classification of samples from the environments. New approaches to ‘denoising’ sequencing data that avoid collapsing OTUs to higher taxonomic levels or *a priori* OTU similarity thresholds, such as ASVs approach (Callahan, 2017), might also further increase the ability to recover the micro-environmental differences among samples.

Although the results show the importance of rare OTUs in discriminating oral subsites and in revealing batch effects, they don’t prove that the rare OTUs are real. Further experimental studies are necessary to provide a direct evidence of their existence. Models of microbial communities where a signal from rare species can be captured and compared with signals from common species would be also helpful to explore rare species and to validate the approach. There are, however, some challenges in developing a realistic model of microbial communities. Available computational tools, such as “SPIEC-EASI” R package (Kurtz et al., 2015) generate a synthetic OTU data using a random selection of species. The randomness contradicts the major assumption of the Anets algorithm that the selection of species in the sample is not random. In addition, the OTU tables simulated by a random selection don’t necessary conform to the occupancy–abundance relationship (Gaston, 1996) observed in real settings.

The transformation of OTU table into the OTU presence/absence values for analysis by Anets places some limitations and constraints on the approach. One such constraint is the presence of many common OTUs, such as found in more than ∼75% samples. The loss of abundance data is another limitation. The information can be important for understanding dominant taxa and their interdependencies with each other and members of the rare biosphere. Another important condition for successful application of the approach is the species co-dependence in the studied environments. The condition is important to observe similar co-occurrence profiles for the associated OTUs and to simplify their clustering. Although this assumption is consistent with known metabolic and functional dependences of microbial species in different environments (Jousset et al., 2017), these dependences are not always the major factors that discriminate environments in a particular study.

Further studies are necessary to validate the proposed framework, to extend it by incorporating additional statistical tools, to provide guidelines on setting parameters for the Anets-Samples and Anets-OTUs, to explore different measures of similarity and their cutoffs, and to clarify limitations of the approach. Further work is also necessary to streamline all calculations in a package. At this point, the computations proposed in the framework are implemented by different programs, such as Anets (Karpinets et al., 2012), Cytoscape (Smoot et al., 2011), mcl (Markov clustering) (Van Dongen, 2008), as well as by simple in-house scripts written in R (see “Operating Procedure to generate Anets” in Supplementary Data Sheet S1). Importantly, the Anets program was implemented for a single processor to cope with a data of small scale and complexity. The program will be slow in processing large OTU tables generated by increasingly complex datasets. It is important to increase scalability of the algorithm by parallelizing independent computation steps and by designing efficient representation of the sparse data for better memory management.

We have thus taken the first initial steps in incorporating the “rare biosphere” of microbial community data and linking their contribution to environmental and phenotypic characteristics via the Anets algorithm. More interesting relationships may be found by this approach as the rate of accumulation of microbial data in different environments continues to increase and the cost of sequencing continues to decrease. We believe that the Anets technique holds unexplored potential for an in-depth analysis of the data. The approach is useful to reveal inherent patterns in the data without *a priori* knowledge of factors influencing the microbial communities as well as to visualize the patterns as networks or maps.

## Materials and Methods

### Mock Dataset

The dataset was generated manually to illustrate the ANETs approach, and represents an oversimplified case of two artificial environments populated by eight hypothetical species. The environments were randomly sampled in 12 locations as described in **Figure 3A** in more detail. The major goal of the dataset was to provide an intuitive illustration of the proposed framework.

### *Populus* Root Microbiome Dataset

The dataset was described by Shakya et al. (2013). It includes 84 samples that represent a combined (fungal and bacterial) microbiome in rhizoshpere (46 samples) and endosphere (38 samples) of 23 mature *Populus deltoids* trees growing in Tennessee (11 trees) and North Carolina (12 trees) taken in May (23 rhizosphere samples and 21 endosphere samples) and in September (23 rhizosphere samples and 17 endosphere samples). Bacterial (16S rRNA) and fungal (28S rRNA) genes from the samples were sequenced to estimate the abundance of fungal and bacterial OTUs and their association with plant phenotypic, genotypic, and environmental parameters. We initially explore abundance–occupancy relationships in the dataset using all rhizosphere and endosphere samples of the study (**Figure 2**) and then focused our further analysis on 46 rhizosphere samples. The OTU table for these samples was processed using the Anets tool in two ways: (1) to build the association network of OTUs, Anets-OTUs, and (2) to build the association network of samples, Anets-Samples. The Anets-Samples was generated using the Pearson correlation as the measure of association for each pair of samples and a *p*-value threshold equal 0.01. The Anets-OTUs was generated using OTUs that occurred in 10 or more samples. This threshold was necessary to reduce time and memory used by the Anets program for processing the data. The *p*-value threshold was set to 0.05. Markov clustering (Van Dongen, 2008) with the inflation value 1.8 was used to cluster the networks, and Cytoscape (Smoot et al., 2011) was used to visualize the networks. Soil properties for samples collected near 23 trees were analyzed using hierarchical clustering. All soil parameters were normalized before the clustering using the average value of the parameter and its standard deviation. The hierarchical clustering of soil samples was performed using Pearson correlation as the similarity metric and centroid linkage as the clustering method. The analysis was implemented using the Cluster 3 program (Eisen et al., 1998). The Java Treeview^2^ was used to visualize the clusters. The ‘vegan’ R package (Dixon, 2003), function ‘capscale,’ was used to calculate variance partitioning the same way as in the initial study (Shakya et al., 2013).

### Human Microbiome Dataset

The dataset was downloaded from the HMP website http://www.hmpdacc.org/HMQCP/. The dataset is based on the analysis of 16S rRNA gene variable regions 1–3 (V13) and includes 2910 samples obtained from 18 different body sites of 180 healthy men and women. Each site was represented by 145–190 samples, except the vagina (87–89 samples). The data is described in more detail by the Human Microbiome consortia publications (Project, 2012). The input for the analysis was the OTU table generated by the project from sequencing reads by the QIIME (Quantitative Insights Into Microbial Ecology) software (Caporaso et al., 2010). The table is comprised of 43140 OTUs and 2910 samples. For the cluster enrichment analysis we used publically available sample metadata, sex of the participant and body site.

The downloaded OTU table was processed using the Anets-Samples algorithm to build the association network of samples. The network was generated using the Pearson correlation as the measure of association for each pair of samples and the *p*-value threshold 0.01. The *p*-values were calculated using a Monte Carlo simulation approach as described before (Karpinets et al., 2012). The network was visualized using edge-weighted (by *p-*value) spring embedded layout in Cytoscape (Smoot et al., 2011). The Anets-OTUs was generated for a subset of 314 skin samples selected by the analysis as significantly associated (clusters with IDs 2, 10, and 16 in **Figure 2**). The OTUs table of the samples was used as input for the Anets-OTUs algorithm with the following parameters: the minimum number of samples per OTU is 15, and a *p*-value threshold is 0.001. The stringent thresholds were important to limit memory use and the processing time for the Anets program. Markov clustering (Van Dongen, 2008) with the inflation value 1.8 was used to cluster the networks, and Cytoscape (Smoot et al., 2011) was used to visualize the networks and the clustering results. An edge-weighted (by *p*-value) spring embedded layout was used for the network visualization.

### Enrichment Analysis

The analysis was used to find samples enriched in each cluster of OTUs in the Anets-OTUs and to find phenotypic or environmental characteristics enriched in each clusters of samples in the Anets-Samples. In both cases the analysis was done using the Fisher’s exact test to examine independence of rows and columns in a two-dimensional contingency table generated by the following algorithms.

We identified samples enriched in the cluster of OTUs (Anets-OTUs) by linking each clustered OTU to the sample and finding those samples that have the greatest representation by OTUs within the cluster. We used the fisher.test() function in R to calculate probability that the number of OTUs representing a sample in the cluster is significantly greater than the number expected by randomly selecting OTUs in the cluster from a set of all associated OTUs, regardless of sample of origin. All associated OTUs were found as a set of unique OTUs associated significantly (*p*-value < 0.05) with at least one other OTU in the Anets-OTUs. We classified the associated OTUs in two ways: if the OTU belongs to the sample or not, and if the OTU belongs to the cluster or not. Using this classification we created the contingency table with the number of the sample’s OTUs in the cluster, the number of associated OTUs in the sample, the number of OTUs in the cluster that are not from the sample, and the number of associated OTUs that are not found in the sample. Because we performed several statistical tests simultaneously on the same data set, *p*-values calculated by the Fisher exact were adjusted using Bonferroni correction.

Specific characteristics (such as soil conditions in the *Populus* rhizosphere dataset or body subsites in the HMP dataset) enriched in the cluster of samples (Anets-Samples) were identified by linking each sample to the characteristics and revealing the characteristics represented by the greatest number of samples within the cluster. We used the Fisher’s exact test to calculate probabilities that number of samples representing a characteristic within the cluster is significantly greater than the number expected by randomly selecting samples into the cluster from a set of all associated samples. In this case the background of the comparison was a set of all associated samples; they were classified for each cluster and each characteristic to create the contingency table as (i) representing the environmental/phenotypic characteristic or not and (ii) belonging to the cluster or not.

### Generating Networks and Their Statistics for Validation

All datasets for validation were downloaded from the HMP website from the link https://www.hmpdacc.org/hmp/HMMCP/ for 16S rRNA amplicon datasets processed by QQIME and the link https://www.hmpdacc.org/hmp/HMQCP/ for datasets processed by MOTHUR software package using a high stringency approach (Schloss et al., 2011). The ‘phyloseq’ R package (McMurdie and Holmes, 2013) was used to download the datasets, to create OTU tables for oral samples for comparisons, to filter OTUs by occupancy, to generate the UUF distances (default parameters) and to produce PCoA plots (distance measure was set to ‘binary’). The Anets-Samples were generated using Pearson correlation as measure of similarity and setting *p*-value threshold to 0.05. The networks were loaded into Cytoscape software, visualized using spring embedded layout without edge weighting and clustered using MCL algorithm by a Cytoscape plugin ‘clusterMaker2’^3^ by setting the inflation value to 2.0. Another Cytoscape plugin ‘Network Analyzer’^4^ was used to explore topology of the networks and to produce their statistics.

## Author Contributions

TK conceived the study. CWS contributed to the preparation, collection, and analysis of the data. JW, AF, CWS, and JZ provided mentoring guidance and advices throughout the study. TK, VG, JW, AF, CWS, and JZ contributed to writing the manuscript.

## Conflict of Interest Statement

The authors declare that the research was conducted in the absence of any commercial or financial relationships that could be construed as a potential conflict of interest.

## Footnotes

1 https://sourceforge.net/projects/Anets/

2 http://sourceforge.net/projects/jtreeview/

3 http://www.rbvi.ucsf.edu/cytoscape/clusterMaker2/

4 http://apps.cytoscape.org/apps/networkanalyzer
