# Supplementary material for: Linking Associations of Rare Low-Abundance Species to Their Environments by Association Networks
Source: Front Microbiol. 2018 Mar 7;9:297. doi: 10.3389/fmicb.2018.00297 (PMC5850922; doi:10.3389/fmicb.2018.00297)
Supplement: Supplementary file 1 [file Data_Sheet_1.pdf]

Supplementary Material

Linking associations of rare low-abundance species to their environments by association networks

Tatiana V Karpinets[1,2*], Vancheswaran Gopalakrishnan[3,4], Jennifer Wargo[1,3], Andrew P. Futreal[1], Christopher W. Schadt[2,5], and Jianhua Zhang[1]

[1] Department of Genomic Medicine, The University of Texas MD Anderson Cancer Center, Houston, TX, USA

[2] Biosciences Division, Oak Ridge National Laboratory, Oak Ridge, TN, USA

[3] Department of Surgical Oncology, The University of Texas MD Anderson Cancer Center, USA, Houston, TX, USA

[4] Department of Epidemiology, Human Genetics and Environmental Sciences, University of Texas School of Public Health, USA

[5] Department of Microbiology, University of Tennessee, Knoxville, TN, USA

*** Correspondence:**
Mailing address: Genomic Medicine, MD Anderson Cancer Center, 1881 East Rd., Houston, TX 77054; Phone: +1 865 250 2006. E-mail: tvkarpinets@mdanderson.org

Supplementary Figures, Tables, and Data Sheets

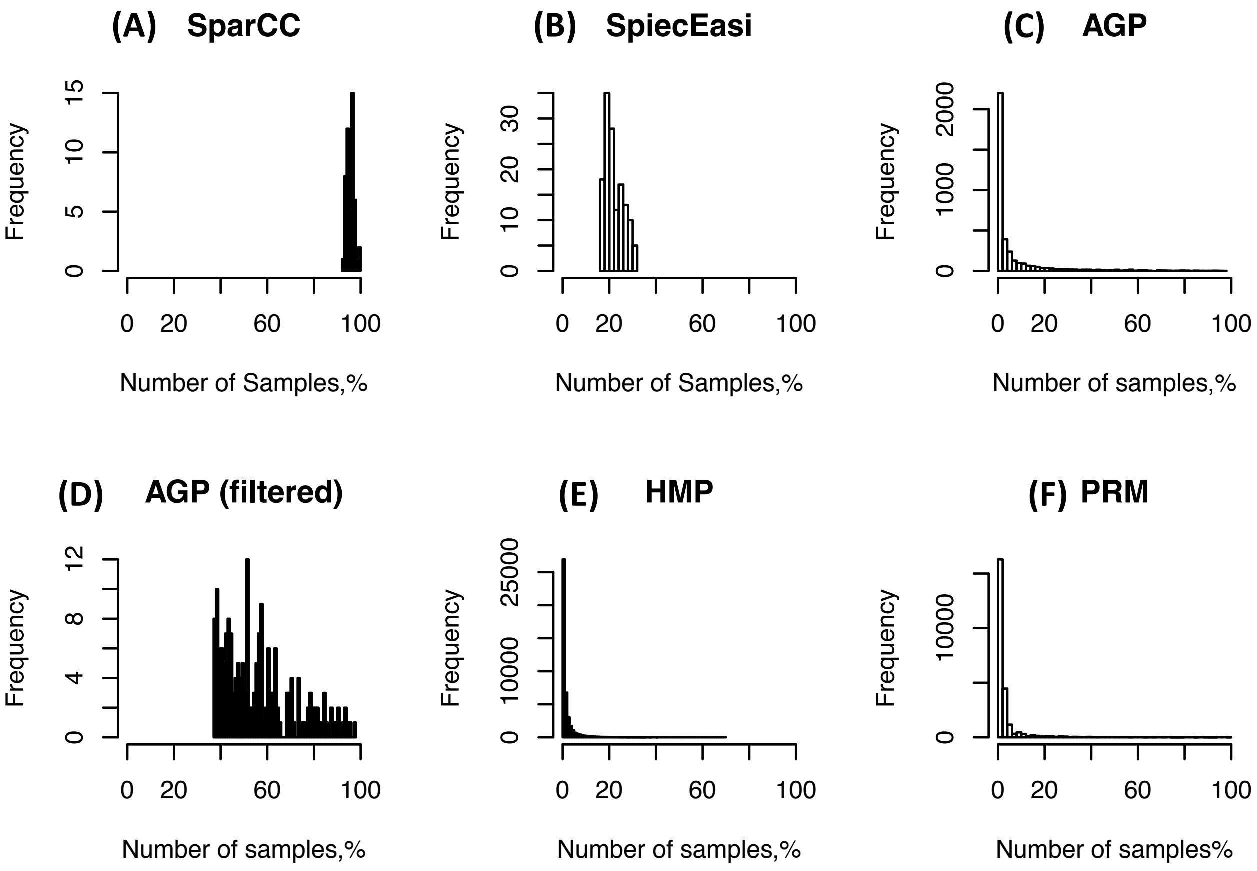

Figure S1. Frequency of OTU as a function of its occupancy calculated as percentage of samples with the OTU from total number of samples: (A) SparCC test dataset; the filtering is the most stringent because the algorithm employs log-transformations of the read counts. **(B)** SpiecEasi benchmark dataset produced from the filtered American Gut Project (AGP) dataset by modeling **(C)** American Gut Project (AGP) dataset unfiltered (3738 OTUs x 407 Samples); **(D)** AGP dataset used to generate SpiecEasi benchmark dataset; the dataset was filtered by removing OTUs that are not found in at least 37% of the samples **(E)** Human microbiome dataset (43140 OTUs x 2910 Samples); 99.9% of OTUs are found in less than 37% of samples **(F)** Populus root microbiome dataset (24434 OTUs x 83 Samples);)about 98% of OTUs are found in less than 37% of samples.

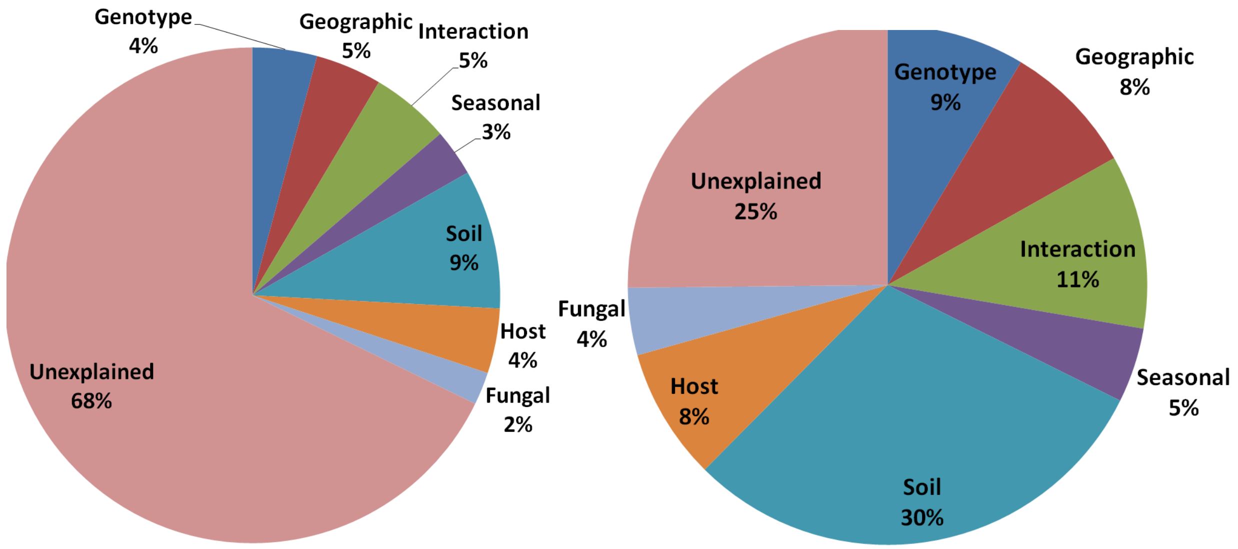

Figure S2. Variance partitioning of bacterial OTUs found in roots of *Populus deltoides* into soil properties, host properties, host genotype, seasonal variability, and beta diversity of corresponding fungal community; all samples from the original study (left) and samples selected by the Anets algorithm as significantly associated (right).

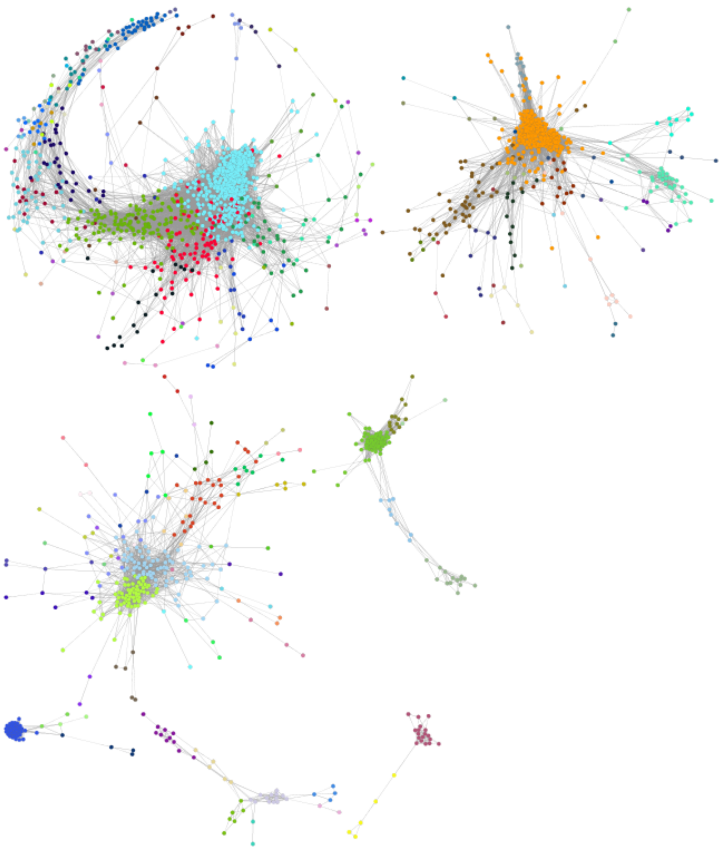

Figure S3. Anets-Samples generated for Human Microbiome Project dataset. Small network components comprised of less then 10 nodes are omitted. The total number of samples (nodes) in the network is 1940. The total number of connections (edges) is 4292. The network was clustered using MCL algorithm (See Method); the same node color indicates the same cluster ID. The total number of clusters is 206.

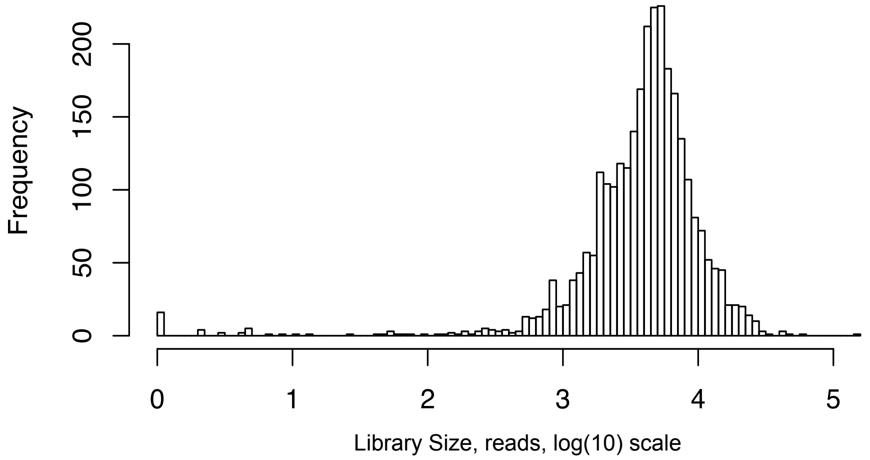

Figure S4. Distribution of the library size values (log10-scale) in oral samples.

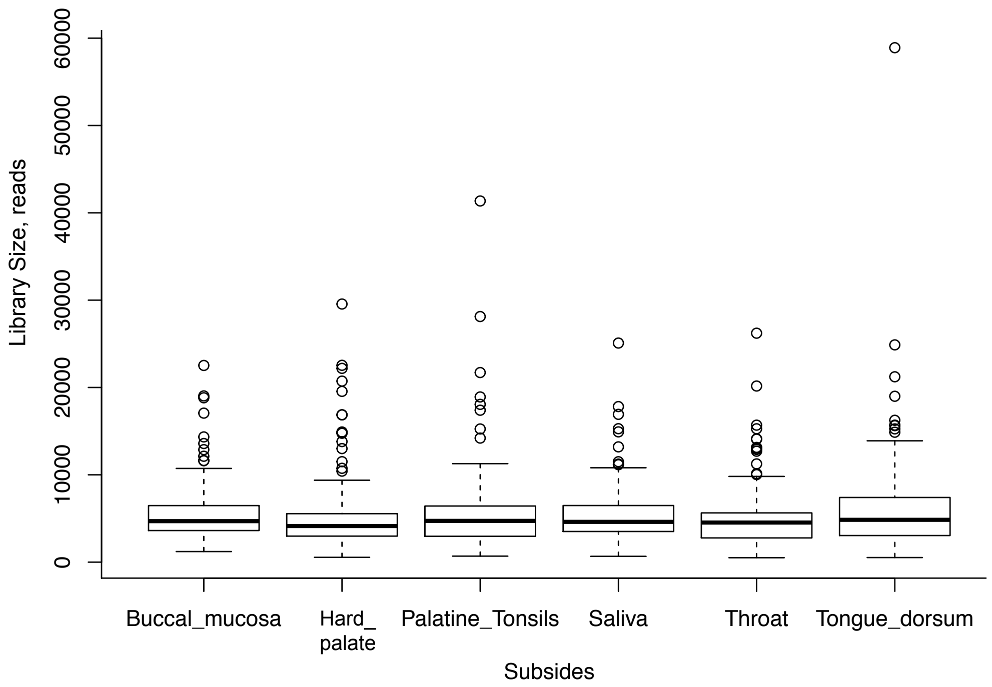

Figure S5. Boxplots of library sizes in different oral subsites.

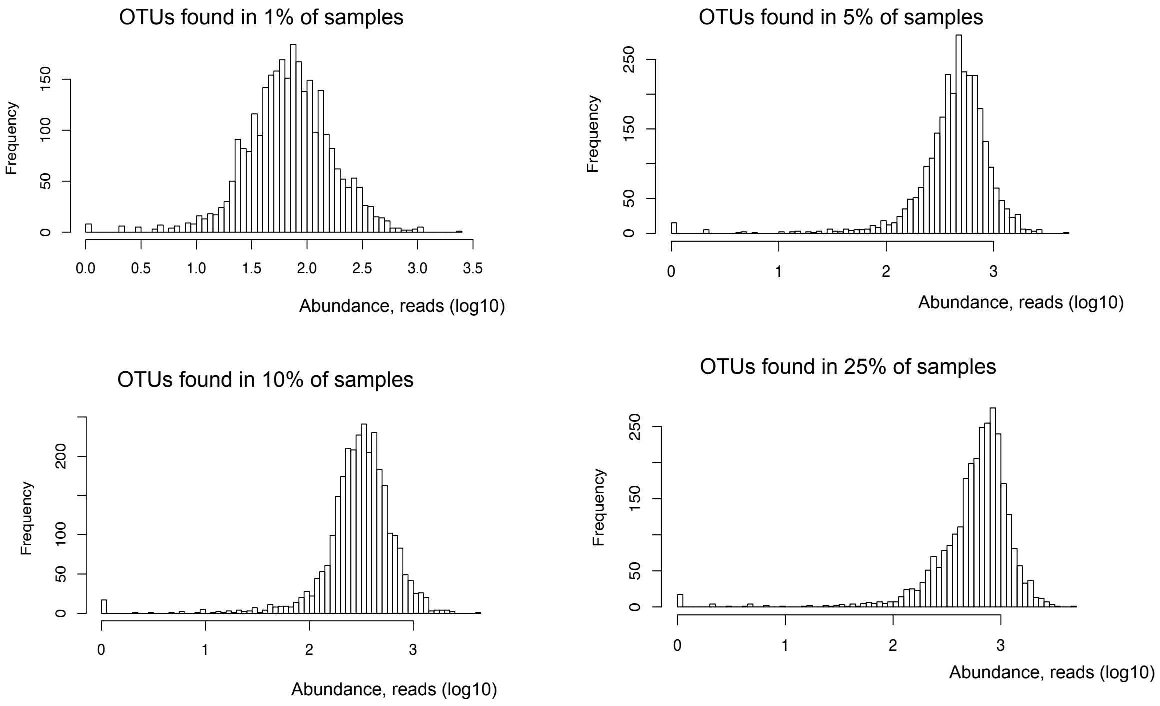

Figure S6. Distributions of abundances of rare OTUs defined by different occupancy thresholds.

(A)

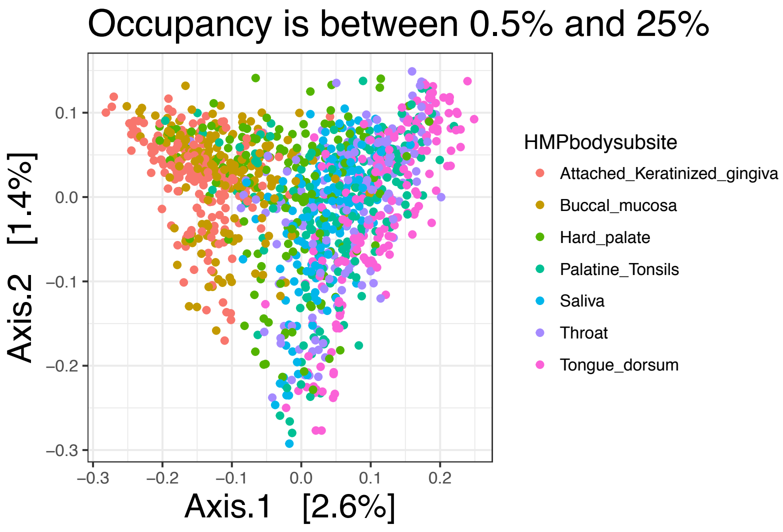

(B)

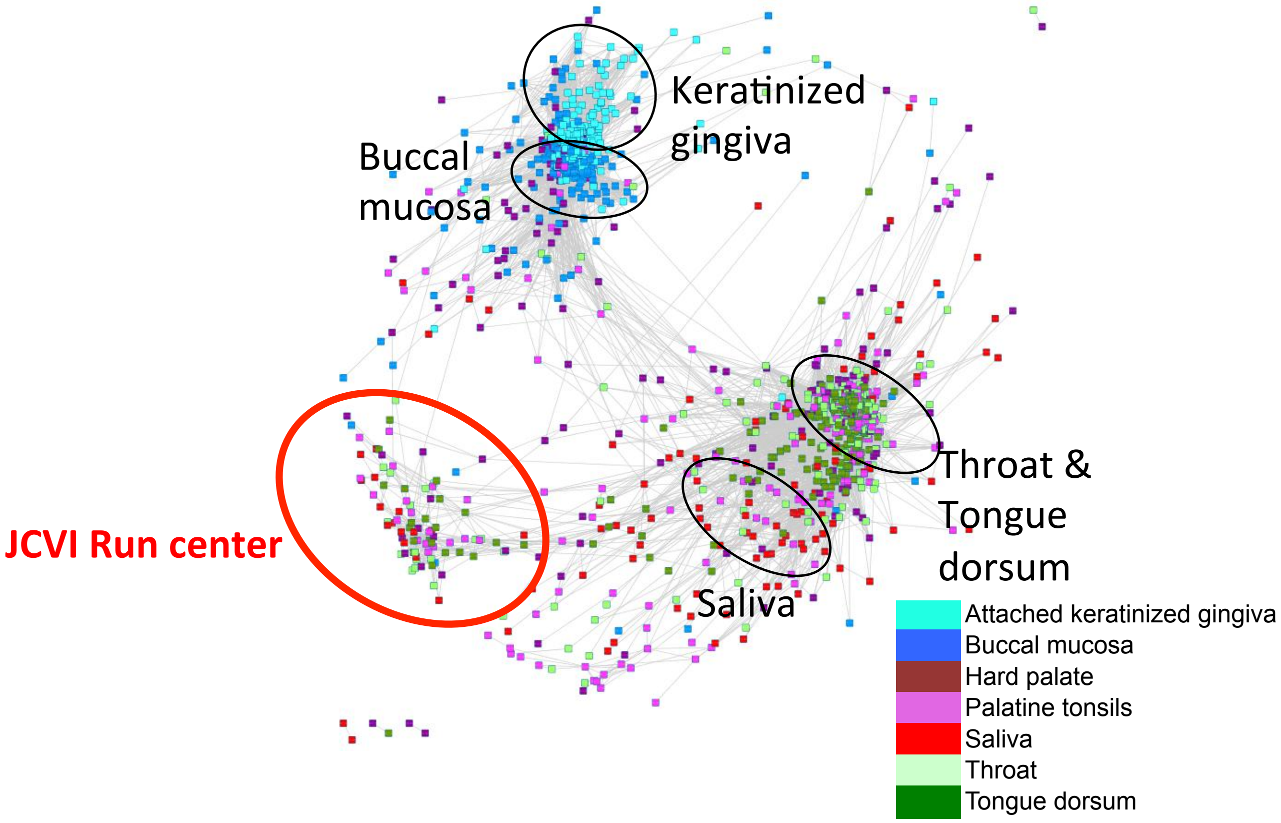

Figure S7. PCoA plot (A) and ANET-samples (B) generated for OTU table comprised of only rare OTUs (occupancy from 0.5% to 25%).

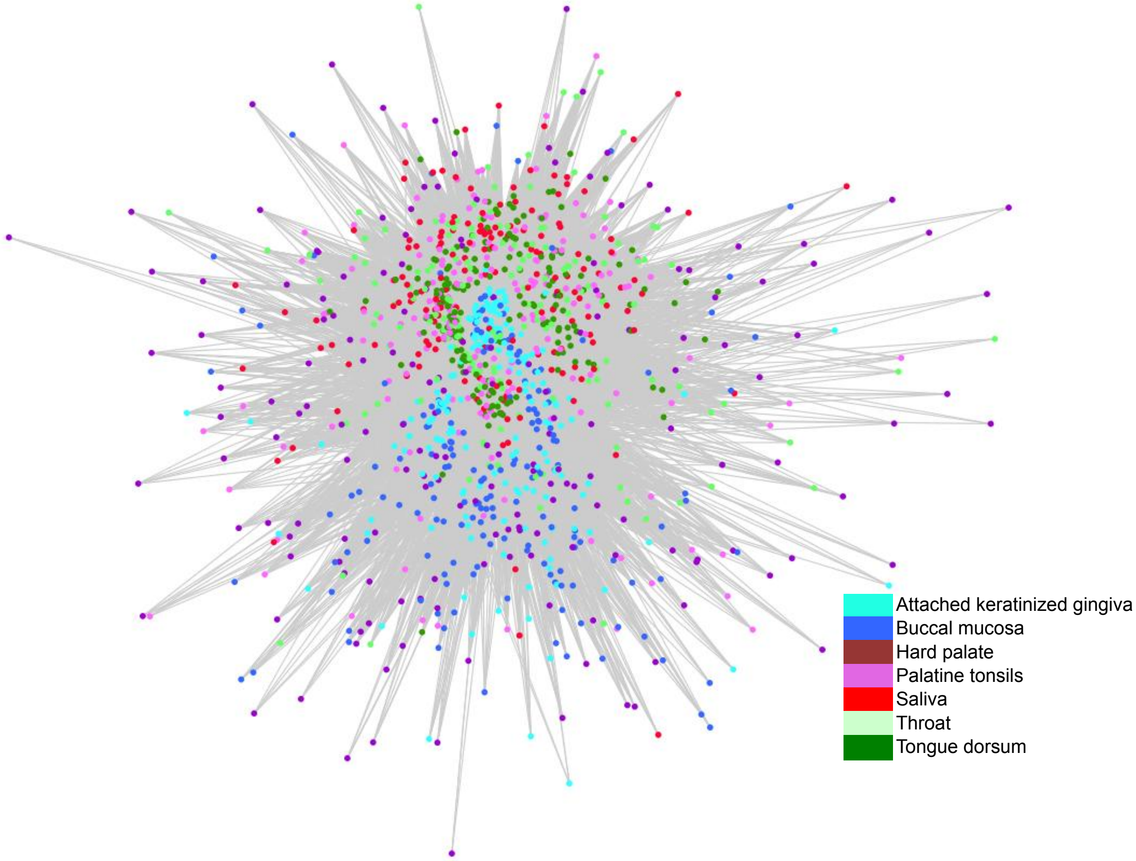

Figure S8. The network of oral samples generated from unweited UniFrac distances (threshold = 0.95).

Table S1. Low taxonomic levels are important to discriminate *Populus* roots microbiomes in TN and NC.

(A) Difference in numbers of associated bacterial species at the level of *Phylum*.

**Kingdom**	**Phylum**	**TN (Cluster2)**	**NC (Cluster3)**
Bacteria	Proteobacteria	30	23
Bacteria	Acidobacteria	24	14

(B) Difference in numbers of associated bacterial species at the level of *Order*.

**Kingdom**	**Phylum**	**Order**	**TN (Cluster2)**	**NC (Cluster3)**
Bacteria	Proteobacteria	Rhodocyclales		4
Bacteria	Proteobacteria	Syntrophobacterales		3
Bacteria	Proteobacteria	Rhodobacterales		2
Bacteria	Proteobacteria	Burkholderiales		1
Bacteria	Proteobacteria	Rhodospirillales	11	2
Bacteria	Proteobacteria	Myxococcales	8	3
Bacteria	Proteobacteria	Rhizobiales	4	3
Bacteria	Proteobacteria	Chromatiales	1	2
Bacteria	Proteobacteria	Proteobacteria	3	2
Bacteria	Proteobacteria	Xanthomonadales	2	1
Bacteria	Proteobacteria	Caulobacterales	1	
Bacteria	Acidobacteria	Sva0725		2
Bacteria	Acidobacteria	Acidobacteria	6	6
Bacteria	Acidobacteria	Acidobacteriales	5	5
Bacteria	Acidobacteria	DS-18	1	1
Bacteria	Acidobacteria	Solibacterales	12	

Table S2. Significant association between microbial communities identified by Anets-OTUs and clusters of samples inferred by Anets-samples.

Microbial community ID in the Anets-OTUs (Figure 5B)	Cluster ID in the Anets-Samples (Figure 5A)	Enrichment of samples labeled by the cluster ID in each microbial community (p<0.01)	Total number of samples in the cluster	%
1	2	255	256	99.61
1	10	40	40	100.00
2	2	93	256	36.33
3	10	36	40	90.00
4	16	18	18	100.00

Table S3. Difference in number of rare OTUs among each pair of subsites. The number of rare OTUs was calculated using 4 thresholds of the occupancy, from more rare (found in 1% of samples, to less rare (found in 5%, 10%, and 25% of samples).

		Mean # of OTUs found in 1% of samples				Mean # of OTUs found in 5% of samples				Mean # of OTUs found in 10% of samples				Mean # of OTUs found in 25% of samples			
**Subsite1**	**Subsite1**	**Subsite1 mean**	**Subsite2 mean**	**p-value**		**Subsite1 mean**	**Subsite2 mean**	**p-value**		**Subsite1 mean**	**Subsite2 mean**	**p-value**		**Subsite1 mean**	**Subsite2 mean**	**p-value**	
**Buccal_mucosa**	**Hard_palate**	53	54	4.3E-01		323	329	4.8E-01		547	546	3.2E-01		852	860	5.6E-01	
Buccal_mucosa	Palatine_Tonsils	53	71	6.6E-05	***	323	377	4.6E-03	**	547	584	2.3E-01		852	875	6.5E-01	
Buccal_mucosa	Saliva	53	61	3.0E-01		323	368	1.0E-01		547	608	1.9E-01		852	940	1.5E-01	
Buccal_mucosa	Throat	53	65	3.1E-03	**	323	356	6.7E-02		547	552	9.4E-01		852	838	4.0E-01	
Buccal_mucosa	Tongue_dorsum	53	78	1.6E-06	***	323	406	7.4E-04	***	547	608	1.5E-01		852	889	5.8E-01	
Hard_palate	Palatine_Tonsils	54	71	2.4E-06	***	329	377	3.5E-04	***	546	584	2.1E-02	*	860	875	2.7E-01	
Hard_palate	Saliva	54	61	6.9E-02		329	368	2.5E-02	*	546	608	3.2E-02	*	860	940	5.3E-02	*
Hard_palate	Throat	54	65	1.3E-04	***	329	356	7.3E-03	**	546	552	2.9E-01		860	838	9.0E-01	
**Hard_palate**	**Tongue_dorsum**	54	78	2.7E-08	***	329	406	9.9E-05	***	546	608	2.3E-02	*	860	889	3.5E-01	
Palatine_Tonsils	Saliva	71	61	8.5E-03	**	377	368	3.7E-01		584	608	7.6E-01		875	940	2.3E-01	
Palatine_Tonsils	Throat	71	65	2.0E-01		377	356	2.3E-01		584	552	1.6E-01		875	838	2.1E-01	
Palatine_Tonsils	Tongue_dorsum	71	78	3.2E-01		377	406	3.7E-01		584	608	6.6E-01		875	889	8.6E-01	
Saliva	Throat	61	65	1.2E-01		368	356	1.0E+00		608	552	1.5E-01		940	838	3.6E-02	*
Saliva	Tongue_dorsum	61	78	6.6E-04	***	368	406	1.2E-01		608	608	9.3E-01		940	889	2.6E-01	
Throat	Tongue_dorsum	65	78	3.8E-02	*	356	406	8.3E-02		552	608	1.4E-01		838	889	2.7E-01	
% significant comparisons				60				40				20				13	

Subsites pairs that have different library sizes are given in bold font.
p-values (Mann-Whitney test) are labeled as follows:

***	0.001
**	0.01
*	0.05

Table S4. Statistics of the networks generated using ANET-samples algorithm and Unweighted UniFrac distance.

Network characteristics	UUF, *D=0.98	UUF, *D=0.95	ANET-samples
Clustering Coefficient	0.04	0.21	0.523
Connected components	1	1	1
Network diameter	5	3	15
Network radius	3	2	8
Network centralization	0.3	0.8	0.2
Shortest paths	752556	1543806	1027182
Characteristic path length	2.8	1.9	4.1
Avg.number of neighbors	14.9	110	53.3
Number of nodes	868	1243	1014
Network density	0.02	0.09	0.05
Network heterogeneity	1.8	1.2	1

*D: Threshold for the UUF (Unwieghted UniFrac) distnace used to generate the network

Table S5. Statistics of the networks generated by ANET-samples algorithm for different OTU tables.

Network characteristics	HMPv13(MOTHUR)	HMPv13(QIIME)	HMPv35(QIIME)	HMPv35(QIIME)Validation
Clustering Coefficient	0.56	0.54	0.54	0.54
Connected components	7	13	9	8
Network diameter	11	19	15	23
Network radius	1	1	1	1
Network centralization	0.2	0.2	0.2	0.2
Shortest paths	849178	1107800	1041440	852894
Characteristic path length	2.9	4.3	4.5	5
Avg.number of neighbors	72.9	71	61	56
Number of nodes	935	1082	1038	943
Network density	0.08	0.07	0.06	0.06
Network heterogeneity	0.94	1.05	1.14	1.07

Data Sheet 1. Operating Procedure to generate Anets

The following programs must be installed on your computer to generate Anets:

1) The Anet program. You can download it at https://sourceforge.net/projects/anets/ . The program is written in C++. You need Linux or MacOS to run it. See `anet-documentation.pdf` file for details.

2) Cytoscape or any other software of your choice on any platform to visualize and cluster the produced network.

The basic workflow to generate Anets-OTU or Anets-Samples is the following

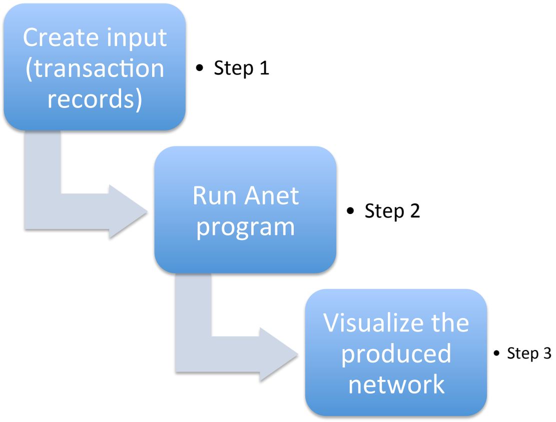

Step 1. Creating the <input file> for the Anet program to generate Anets-OTUs and Anets-Samples

The <input file> for the Anet program is a text file with only one column with a transaction record in each row. The transaction record is just a list of items that goes together, such as a list of OTUs found in each samples. For OTU table (`OTUtable_SimulatedStudy.txt)` produced in the simulated study, which looks like

Species	Sample 1	Sample 2	Sample 3	Sample 4	Sample 5	Sample 6	Sample 7	Sample 8	Sample 9	Sample 10	Sample 11	Sample 12
**E1_red**	**1**	**1**	**1**	**0**	**0**	**0**	**0**	**0**	**0**	**0**	**0**	**0**
**E1_blue**	**1**	**0**	**0**	**1**	**0**	**1**	**0**	**0**	**0**	**0**	**1**	**0**

**E1_green**	**0**	**1**	**0**	**1**	**1**	**0**	**0**	**0**	**0**	**0**	**0**	**0**
**E1_brown**	**0**	**0**	**1**	**0**	**1**	**1**	**0**	**0**	**0**	**0**	**0**	**0**
**E2_red**	**1**	**0**	**0**	**0**	**0**	**0**	**1**	**0**	**0**	**1**	**0**	**0**
**E2_blue**	**0**	**0**	**0**	**0**	**0**	**0**	**0**	**1**	**1**	**0**	**1**	**1**
**E2_green**	**0**	**0**	**0**	**0**	**0**	**0**	**0**	**1**	**0**	**0**	**1**	**0**
**E2_brown**	**0**	**1**	**0**	**0**	**0**	**0**	**1**	**0**	**1**	**1**	**1**	**1**

the input file (`OTUtable_SimulatedStudy_tr_OTUs.tr`) to generate Anets-OTUs must look like

```
E1_red E1_blue E2_red

E1_red E1_green E2_brown

E1_red E1_brown

E1_blue E1_green

E1_green E1_brown

E1_blue E1_brown

E2_red E2_brown

E2_blue E2_green

E2_blue E2_brown

E2_red E2_brown

E1_blue E2_brown E2_blue E2_green

E2_blue E2_brown
```

The input file (`OTUtable_SimulatedStudy_tr_samples.tr`) to generate Anets-Samples must look like

```
Sample.1 Sample.2 Sample.3

Sample.1 Sample.4 Sample.6 Sample.11

Sample.2 Sample.4 Sample.5

Sample.3 Sample.5 Sample.6

Sample.1 Sample.7 Sample.10

Sample.8 Sample.9 Sample.11 Sample.12
```

```
Sample.8 Sample.11

Sample.2 Sample.7 Sample.9 Sample.10 Sample.11 Sample.12
```

Below is a simple R script to generate input files for Anets-OTUs and Anets-Samples for the OTU table given above. The OTU table file `OTUtable_SimulatedStudy.txt` (tab-delimited) was loaded from the directory `"/Users/tvkarpinets/Documents/ANET/"` on the mac computer. You will need to use '\' instead of '/' if you have Windows.

```
>my_OTU=read.delim("/Users/tvkarpinets/Documents/ANET/OTUtable_SimulatedStudy.tx
t", row.names="Species", header=TRUE, sep='\t')

>my_tr_samples=c; for (i in 1:length(rownames(my_OTU))) { p1=
as.vector(unlist(my_OTU[i,]));
my_tr_samples=rbind(my_tr_samples,paste(colnames(my_OTU)[p1!=0], collapse=" "))}

> write(my_tr_samples,
file="/Users/tvkarpinets/Documents/ANET/Anets_Samples_input.tr")

>my_tr_otus=c()

>for (i in 1:length(colnames(my_OTU))) { p1= as.vector(unlist(my_OTU[,i]));
my_tr_otus=rbind(my_tr_otus,paste(rownames(my_OTU)[p1!=0], collapse=" "))}

>write(my_tr_otus, file="/Users/tvkarpinets/Documents/ANET/
Anets_OTUs_input.tr")
```

Step 2. Run the Anet program to generate Anets-OTUs and Anets-Samples

By default, the Anet program will be installed on your computer in the directory 'anet-v1.0', and the Anet program executable file will be in the directory 'bin', inside the folder *'anet-v1.0'*. You can copy your transaction files (`Anets_OTUs_input.tr` and `Anets_Samples_input.tr`) generated at the previous step into the 'bin' directory and run the program from the bin directory after you change permissions (chmod +x anet) as

anet --file=<input file> --method=<default:spearman> --by=<by_item_count (default) or by_cooccur_count> --threshold=<default:1.0> --count=<count value> --output=<output file name>

where

- input file
  input file name (or full path)

-- correlation type
  spearman (default), pearson, or cosine

- output file
  output file name (or full path)

- filtering method
  This option is used to filter out annotations whose occurrences in the data
  do not show statistical significance. Currently the following two options
  are supported. In both cases, the threshold value is specified by "--count" option.

1. by_cooccur_count (default)
2. by_item_count

- count
  This option is used to specify the threshold value for the filtering method.
  The default is one.

- threshold
  An input to this option is the largest p-value of the output entries,
  i.e. pairs of annotations.

Parameters of the program depend on characteristics of the OTU table (how large the table and how sparse it is). The Anet program may be slow in processing large OTU tables as discussed in Methods. In this case you may need to limit the number of OTUs or samples for the analysis. It can be done by 2 parameters in the Anet program (--by and –count).

For medium size OTU table, such as 20000 OTUs x 80 Samples, we recommend to set the following initial parameters when generating Anets-OTUs:

--method=pearson
--by=by_cooccur_count
--count= 10 (you may increase this value if the generated network file is large)
--threshold=0.05

For Anets-Samples, you may need only a threshold for p-value because as a rule the total number of Samples is essentially less than the number of OTUs:

--method=pearson
--threshold=0.05 (or 0.01)

Below are examples how to run Anet for the transaction-files produced for the simulated study and given above:

```
./anet --file=OTUtable_SimulatedStudy_tr_OTUs.tr --method=pearson --by=by_cooccur_count  --count=1 --
threshold=1  --output=OTUtable_SimulatedStudy_tr_OTUs_out.txt

./anet --file=OTUtable_SimulatedStudy_tr_samples.tr --method=pearson --by=by_cooccur_count  --count=1 --
threshold=1  --output=OTUtable_SimulatedStudy_tr_samples_out.txt
```

Output of the program is a text file of the network with 5 columns. Each row shows characteristics of the edge in the network. The first two columns are connected nodes and the rest are characteristics of the connection that include the association coefficient (in our case it will be the Pearson correlation) and its significance (p-value).

Example for the simulated study is given below:

```
./anet --file=OTUtable_SimulatedStudy_tr_OTUs.tr --method=pearson --by=by_cooccur_count  --count=1 --
threshold=1  --output=OTUtable_SimulatedStudy_tr_OTUs_out.txt

./anet --file=OTUtable_SimulatedStudy_tr_samples.tr --method=pearson --by=by_cooccur_count  --count=1 --
threshold=1  --output=OTUtable_SimulatedStudy_tr_samples_out.txt
```

In this case we output all edges and filter later by the correlation coefficient (R=0.30), because p-values generated by a Monte Carlo simulation will not make sense for a small set.

The Anets-OTUs filtered by R=0.30 will look like

Annotation1	Annotation2	Correlation	p-value	#Records(Anno1,Anno2)	#Records(Anno1)	#Records(Anno2)	#CoAnnos(Anno1)	#CoAnnos(Anno2)
E2_blue	E2_green	0.8664	0	2	4	2	3	3
E2_brown	E2_blue	0.68299	0.0357143	3	6	4	6	3
E2_red	E2_brown	0.462708	0.0714286	2	3	6	3	6
E1_green	E1_brown	0.382971	0.107143	1	3	3	4	3
E2_brown	E2_green	0.320604	0.142857	1	6	2	6	3
E1_red	E1_green	0.3114	0.178571	1	3	3	5	4
E1_red	E1_brown	0.298142	0.214286	1	3	3	5	3

See anet-documentation file for description of the columns.

For large OTU table, with many samples, we recommend to start the analysis by generating the Anets-Samples with different p-value cutoffs and set –method = pearson. The other parameters may be set to default values. In our experience, the proper filtering by p-value is important to generate a biologically meaningful network with optimal resolution and remove noisy samples. This will also simplify further clustering of the network. If the generated network file is very large you may consider filtering the network file by selecting only edges with p-value less than 0.01, 0.005 and so on. The best way to see if the network reproduces environment of your study is to visualize it and overlay with the metadata as described in Step 3. If you see that clusters of samples in the network correspond to your metadata, you may consider further analysis using Anets-OTUs. In this case, however, you may have to introduce additional thresholds for the analysis to limit the number of OTUs for processing and the number of samples, because the Anet program will quickly increase the processing time when you increase the number of unique OTUs in the input.

Step 3. Anets visualization and clustering

There are many ways you can visualize the generated network. Many languages include packages or modules to work with networks, and it is always better to use the programming language you are most familiar with. One easy way to visualize, analyze, and cluster the network as well as explore its properties is by using Cytoscape (http://www.cytoscape.org/). This software is designed for complex network analysis and visualization, and it is freely available for different platforms. The documentation is available at https://bix-lab.ucsd.edu/display/Public/Cytoscape+3.2.X+Visualization+and+Analysis+Documentation. The file generated by Anet program can be easily imported into the software as the network file. Use options in the File menu as File> Import>Network>File>. This selection will open the window where you need to select the "Source Interaction" as Column 1 and the "Target Interaction" as Column 2. You should leave the "Interaction Type" as Default. You may import the rest columns as edge (interaction) annotations by clicking on each of them and to use the correlation coefficient or p-values for filtering the network or, as weights, for visualization. After you import the network you may visualize it using one of the provided layouts. In our experience a nice visualization can be obtained using a spring embedded

layout. Importantly, the software allows you to load the metadata file of the samples (for Anets-Samples) or of the OTUs (for Anets_OTUs). You can use an option File> Import>Table>File. After you import the metadata, you can easily color nodes of the network according to the annotations and to see if the obtained results are meaningful. You can easily cluster the network using Cytoscape as well. To do this you need to install additional plugin called 'clusterMaker2' using App Manager (Apps> App Manager). A diverse set of clustering options available in this plugin including the Markov clustering algorithm and the Community Clustering algorithm, both showed a good performance in our experience. You can export the clustering results with the metadata as a comma separated text file using an export options in the File menu (File> Export>Table>clustered default node>).
